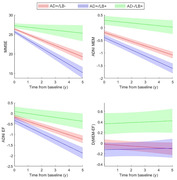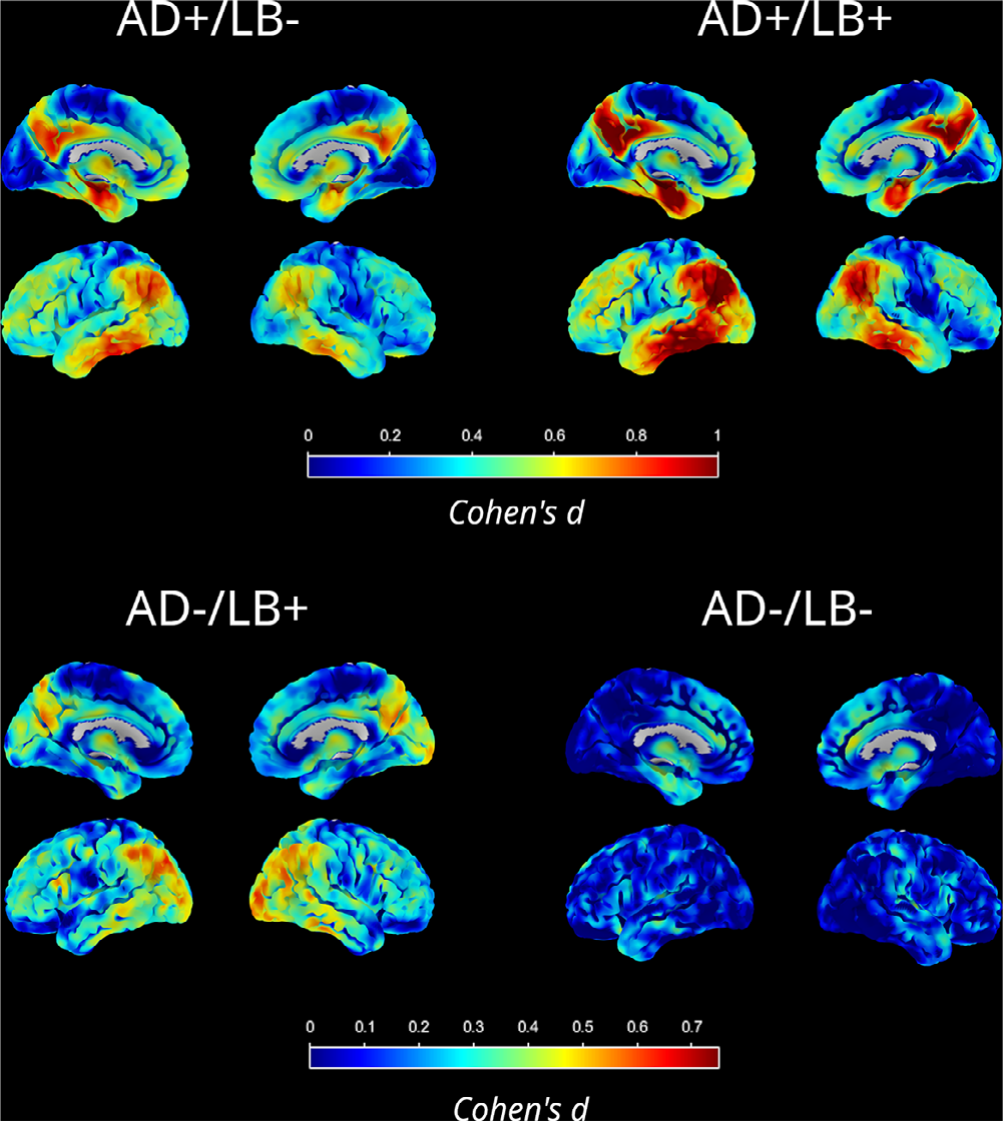# Neurobiological and clinical characteristics of amnestic patients with Lewy body pathology

**DOI:** 10.1002/alz.088826

**Published:** 2025-01-09

**Authors:** Jesús Silva‐Rodríguez, Alexis Moscoso, Miguel Labrador‐Espinosa, Pascual Sanchez‐Juan, Michael Schöll, Michel J. Grothe

**Affiliations:** ^1^ Reina Sofia Alzheimer Center, CIEN Foundation, ISCIII, Madrid, Madrid Spain; ^2^ Wallenberg Centre for Molecular and Translational Medicine, University of Gothenburg, Gothenburg Sweden; ^3^ Reina Sofia Alzheimer Centre, CIEN Foundation, ISCIII, Madrid Spain

## Abstract

**Background:**

While Lewy body (LB) pathology typically associates with a distinct clinical profile compared to Alzheimer’s disease (AD), early memory deficits are not uncommon and can confound clinical diagnosis of amnestic patients. Moreover, approximately 30‐60% of AD patients have concomitant LB pathology, which has been reported to affect the clinical phenotype and result in a more aggressive disease course. Recently developed α‐synuclein seed amplification assays (αSyn‐SAAs) have demonstrated high diagnostic accuracy for LB diseases, but the role of these novel biomarkers in the context of amnestic presentations typical for AD remains to be investigated in more detail.

**Method:**

We studied 872 patients from the ADNI cohort with a baseline diagnosis of aMCI (N=661) or AD dementia (N=211), who had CSF and FDG‐PET data available. CSF samples were analyzed for peptide levels of Aβ1‐42 and p‐tau181, and αSyn positivity was evaluated using a novel αSyn‐SAA. Based on positive/negative results on the different biomarkers, subjects were grouped into “AD‐/LB‐” (A‐T‐αSyn‐, N=106), “AD+/LB‐” (A+T+αSyn‐, N=336), “AD+/LB+” (A+T+αSyn+, N=158) and “AD‐/LB+” ((A‐|T‐)αSyn+, N=68). We analyzed group differences in demographics, APOE4 positivity, neurodegeneration patterns (FDG‐PET), as well as baseline and longitudinal scores of global cognition (MMSE) and domain‐specific function (ADNI_MEM, ADNI_EXEC).

**Result:**

At baseline, AD+/LB+ showed worse global cognition (MMSE: d=‐0.27, p=0.004) and memory performance than AD+/LB‐ (ADNI_MEM: d=‐0.26, p<0.006). AD‐/LB+ were less globally impaired (p<0.001) and characterized by a markedly more dysexecutive profile (d(MEM‐EXEC): p<0.002). AD+/LB+ progressed faster on all cognitive scores, but they did not develop a differential cognitive profile compared to AD+/LB‐ (Figure 1). APOE4 positivity was similar between AD+/LB+ and AD+/LB‐ (72% vs. 75%, p=0.28) but lower in AD‐/LB+ (28%, p<0.001). In FDG‐PET, AD+/LB+ was characterized by a temporo‐parietal hypometabolism pattern similar to that of AD+/LB‐ but with higher effect sizes (Fig‐2). By contrast, AD‐/LB+ showed a strikingly different posterior‐occipital pattern of hypometabolism.

**Conclusion:**

LB co‐pathology in AD was associated with more severe neurodegeneration and faster cognitive decline, but an amnestic AD‐typical phenotype. By contrast, patients with relatively pure LB pathology were characterized by a more dysexecutive cognitive profile and a distinct hypometabolism pattern typically suggestive of LB disease.